# Classification of Hemodynamics Scenarios from a Public Radar Dataset Using a Deep Learning Approach

**DOI:** 10.3390/s21051836

**Published:** 2021-03-06

**Authors:** Gašper Slapničar, Wenjin Wang, Mitja Luštrek

**Affiliations:** 1 Department of Intelligent Systems, Jožef Stefan Institute, 1000 Ljubljana, Slovenia; mitja.lustrek@ijs.si; 2 Jožef Stefan International Postgraduate School, 1000 Ljubljana, Slovenia; 3 Department of Electrical Engineering, Eindhoven University of Technology, 5600 MB Eindhoven, The Netherlands; wwang@tue.nl or; 4 Philips Research Eindhoven, 5656 AE Eindhoven, The Netherlands

**Keywords:** contact-free sensing, radar data, classification, deep learning, artificial neural networks

## Abstract

Contact-free sensors offer important advantages compared to traditional wearables. Radio-frequency sensors (e.g., radars) offer the means to monitor cardiorespiratory activity of people without compromising their privacy, however, only limited information can be obtained via movement, traditionally related to heart or breathing rate. We investigated whether five complex hemodynamics scenarios (resting, apnea simulation, Valsalva maneuver, tilt up and tilt down on a tilt table) can be classified directly from publicly available contact and radar input signals in an end-to-end deep learning approach. A series of robust k-fold cross-validation evaluation experiments were conducted in which neural network architectures and hyperparameters were optimized, and different data input modalities (contact, radar and fusion) and data types (time and frequency domain) were investigated. We achieved reasonably high accuracies of 88% for contact, 83% for radar and 88% for fusion of modalities. These results are valuable in showing large potential of radar sensing even for more complex scenarios going beyond just heart and breathing rate. Such contact-free sensing can be valuable for fast privacy-preserving hospital screenings and for cases where traditional werables are impossible to use.

## 1. Introduction

Wearable sensors have achieved wide popularity and are being used daily by people to monitor a variety of things, ranging from their day-to-day activities to their overall health [1]. This is a consequence of many advantages of these devices, such as their small form-factor (e.g., wristbands), relatively low cost and general ease of use. These advantages allow for many different user groups to use wearables regularly [2], however, wearable sensors are not ideal, and there are still cases where usage is not optimal or even possible. Examples where limitations become apparent are for instance sleep monitoring (due to discomfort of wearing a sensor during sleep), monitoring of people with dementia (as they can quite possibly forget to charge the battery or even equip the device itself), or any prolonged usage where battery constraints become a concern. Additionally, in a clinical emergency setting, speed at which measuring is available for different patients can also be an important factor—for instance, the ongoing coronavirus disease (COVID-19) pandemic has pushed many hospitals to the limit, and the time needed to conduct a common respiratory checkup with traditional methods is large compared to a contact-free setup, which can be available continuously and the patient just placed in front of it without notable set-up time [3].

The aforementioned challenges have spurred researchers to focus their attention towards potential contact-free sensor alternatives. Optical sensors (especially cameras) offer the means to capture rich information about the user, including their activity and physiological information. The latter is most commonly obtained via remote photoplethysmography (rPPG), which allows for computation of physiological parameters such as heart rate (HR) and heart rate variability (HRV), which are in turn known to be connected to mental states, such as deterioration, stress or cognitive load. There are however downsides to using optical sensors, such as sensitivity to environmental conditions (e.g., lighting), privacy intrusion (as a person can be identified on camera) and difficulty in ensuring consistent skin exposure, as skin visibility can be limited by many common things, such as facial hair, glasses, make up, etc. To combat these issues, some research investigated radio frequency-based (RF-based) contact-free sensors, to obtain similar valuable physiological parameters, such as HR and respiration rate (RR). Such sensors offer better privacy protection and show promising results in physiological data analysis, even when user exposure is not optimal in cases of occlusions or barriers. We discuss existing research work in the field of contact-free sensors in more detail in the following section.

In our work we focused on analysis of radar contact-free data and comparison between contact and contact-free data (and their fusion) for classification of several hemodynamics scenarios. In these scenarios, the blood flow of a person is being influenced by some experimental protocol activities (e.g., tilt of the body on a tilt table, execution of the Valsalva maneuver, simulation of apnea), which is expected to cause the person to exhibit physiological changes related to their cardiovascular and respiratory state. Since we had both data modalities (contact and contact-free) available, we investigated if the contact-free sensor data can achieve similar performance to traditional contact sensors for such classification, thus being able to capture similar level of information. Furthermore, we investigated whether the multi-modal data fusion can additionally improve the results. The answers to these questions are important, as they help us discern if RF-based contact-free sensors (in this case radar) can actually achieve comparable performance to a traditional hospital contact setup, thus allowing for unobtrusive and privacy-preserving monitoring of patients and detection of their physiological changes. Furthermore, as we are classifying specific scenarios and not just predicting the physiological parameters, we investigated the possibility of an end-to-end classification of complex states, where physiological parameters are not explicitly given or computed. Finally, since we used a publicly available dataset in our research, these results also show the baseline deep learning (DL) classification performance on these data, and can be used as a starting point and a baseline for further research on these data. Such a model for detection of hemodynamics scenarios can be directly useful for apnea detection at home or in a hospital, but also potentially useful for detection of anomalous or otherwise interesting physiological phenomena at home or in a hospital during clinical examinations where speed, patient comfort and anonymity are important.

The rest of this paper is organized as follows: we initially overview related work in the field of contact free sensing for physiological parameters, critically reviewing the advantages and disadvantages of each method. In Section 2, we first present in detail the dataset used in our research. Data preparation and preprocessing are then described and the experimental setup is explained. Finally we report on investigation of different DL architectures. In Section 3, we report the results and in Section 4 we discuss the findings and importance of our study. We conclude in Section 5 with a summary and plans for future work.

### 1.1. Related Work Using Contact-Free Sensors

As highlighted previously, contact-free sensors have some important advantages over traditional contact ones, thus spurring a lot of research in the past decade or so [4]. Cardiorespiratory monitoring has been the major focus and different methods have been been investigated, ranging from optical methods based on the visible light in the electromagnetic spectrum (red, green, blue—RGB) to RF-based methods using radars and WiFi waves, which are in the microwave and radio part of the spectrum.

Since our aim is to classify distinct hemeodynamics scenarios (in which HR and RR potentially change) using only a radar sensor, we focus on related work dealing especially with breathing, although an estimation of heart sound is also available from the data. However, the former is relatively robust and stable while the latter is much more volatile and artefact-prone. Even though photoplethysmogram (PPG) is not being reconstructed and used in this work, we still briefly review rPPG methods, since they are an important part of the contact-free sensor landscape, and in order to highlight the advantages and disadvantages of using RF-based sensors (e.g., radar) vs. optical sensors (e.g., camera or photodetector).

#### 1.1.1. Optical Methods

The first group of methods relies on optical sensors, mainly in the form of different cameras, although low resolution photodetectors are also becoming increasingly interesting for research. Some research also investigated thermal and laser-based methods. The idea is typically to capture the skin tissue and detect either color changes, slight movements, or temperature variations that reflect the cardiorespiratory activity.

##### RGB Camera Color-Based Methods

**Figure 1:**
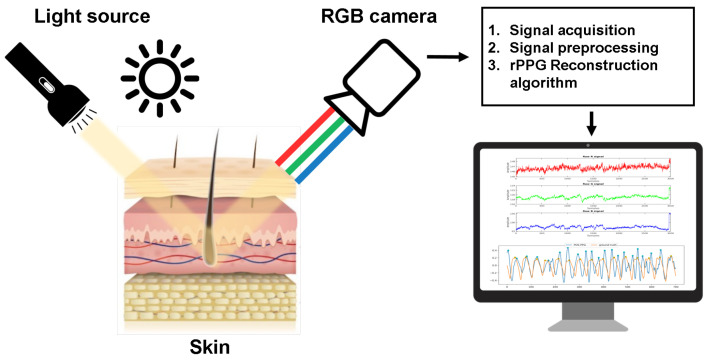
Schematic representation of obtaining physiological signals with an RGB camera. Similar approach is applied using traditional contact wearables.

PPG can be reconstructed using an RGB camera as a photodetector and by either having a dedicated consistent light source or using the ambient light, as shown in Figure 1. This allows for estimation of both HR and RR.

In a light-controlled environment, some research [5,6,7,8,9,10,11,12,13] has succeeded in extracting PPG data based on simple and computationally inexpensive analysis of the red, green and blue channels captured by an RGB video camera. Takano et al. [14] have averaged the pixel values of a single color channel for a specified region of interest (ROI) on the face of participants, who were seated in front of a camera. They used autoregressive spectral analysis to determine frequency peaks, attributing them to HR and respiratory rate. Verkruysse et al. [15] used pixel averages of all three channels of ROI in a fast Fourier transform (FFT) analysis, to obtain dominant frequencies, again corresponding to HR and respiratory rate. Some of the most popular and widely-adapted rPPG methods for HR estimation are a motion robustness method based on chrominance (CHROM) signals by de Haan and Jeanne [16], an rPPG method based on spatial subspace rotation (2SR) proposed by Wang et al. [17], and a plane orthogonal to skin (POS) based-method again developed by Wang et al. [18]. For details on these, we refer the reader to the original papers.

##### RGB Camera Motion-Based Methods

Many researchers have analyzed video and image sequences to detect slight motions of the chest or other body part motions in order to extract physiological signs. Early studies focused on tracking the pixels using optical flow. For example, respiratory activity was estimated by detecting optical flow of the body surface movement resulting from respiratory rhythms by Nakajima et al. [19] and Frigola et al. [20].

Optical flow based methods are heavily affected by artificial noise due to subject movement and difficult ROI tracking. Some researchers thus investigated an alternative in the form of 3D cameras (e.g., Kinect sensor) which capture depth information in some ROI, typically chest or abdomen area [21,22,23,24].

In regards to cardiac activity, slight head movements are caused by the blood being pushed through the carotid artery by the heart. These movements can again be extracted using independent component analysis (ICA) [25], principal component analysis (PCA) [26,27] or a frame subtraction method [28]. Video magnification methods like Eulerian video magnification were also investigated to extract the cardiac pulse signal in different ROIs (e.g., wrist, hand neck and leg) by He et al. [29] and Al-Naji et al. [30].

##### Laser-Based Methods

Continuous monitoring of patients in terms of RR and HR has also been investigated using lasers, specifically a method called laser Doppler vibrometry (LDV). The idea is to use a laser light source with a specific wavelength, which reflects from the surface and is used to determine vibration amplitude and frequency. These can be computed from the Doppler shift of the reflected laser beam frequency due to the motion of the surface.

Scalise et al. [31] used this to measure RR in preterm infants. They measured the abdominal wall movements on a total of 9 preterm infants for 60 s each and have shown high correlation with ground truth respiration measurement. Sirevaag et al. [32] obtained data from 32 participants during resting and then used LDV to detect breathing cycles and heart rate, while analyzing the obtained waveform in detail. High correlation with ground truth circumferential respiratory belt was reported for breathing, and heart activity in terms of HR and inter-beat intervals (IBI) was also well-correlated with ground truth ECG. Chen et al. [33] proposed detection of vibrations on the skin above carotid artery related to arterial wall movement using LDV. They used PCA method to extract the cardiovascular signal and evaluated their method on 285 individuals. Signal was compared with ground-truth ECG using a metric called equal error rates (EER) and they achieved the best performance of 6.3% in intersession testing. Zhang et al. presented an approach to sense human activities across rooms using long-range laser vibrometry in combination with a low resolution CMOS sensor, which captured a retroreflected speckle pattern at a given point. They showed high accuracies in activity recognition (e.g., tool usage and exercise) at long range even in noisy environments [34].

#### 1.1.2. RF-Based Methods

**Figure 2:**
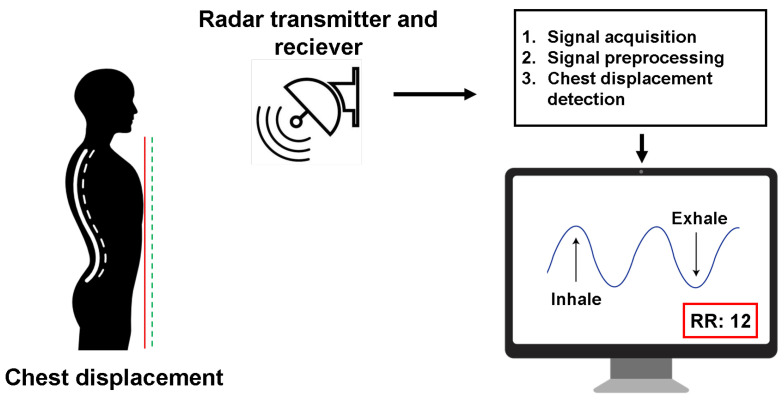
Schematic representation of obtaining physiological signals with a radar.

The second group of methods relies on radio frequency waves and their interaction with the body in order to detect subtle movement. For instance, the chest wall (and to some extent the abdominal area) exhibits such movement due to mechanical activity of the heart and lungs, causing a slight displacement, which can be measured via the Doppler effect using a radar, as shown in Figure 2.

Measurement of human physiological parameters with a radar has been explored with three types of radar systems: continuous-wave, frequency-modulated (FM) and ultra-wide band (UWB). Studies differ in using Doppler radar at different frequencies and distances, but almost all focus on the chest area. An early attempt by Greneker et al. focused on the chest region at a distance of 10 m [35] to obtain HR and respiratory information. Other work followed by using radars at different ranges from roughly 1 m to 10 m, and with different frequencies ranging from roughly 1 GHz to over 80 GHz [35,36,37,38].

Cardillo et al. [39] proposed a new radar sensing approach for automatic detection and ranging of human targets in cluttered environments, without the need for any target thresholding. Their approach is robust to artefacts due to presence of multiple objects within the same radiation beam. Humans are detected via their breathing patterns and separated from non-human targets. Su et al. [40] developed a prototype system for RR and HR monitoring of multiple humans using a single-conversion stepped-frequency continuous-wave radar. It combines a stepped-frequency continuous-wave radar and a self-injection-locked radar to benefit from the range resolution and the Doppler sensitivity of the two radars. They demonstrate feasibility of monitoring vital signs of up to three humans at a time. Su et al. [41] then upgraded their approach by presenting a combination of a self-injection-locked radar with frequency-modulated continuous-wave and switched phased-array techniques used to locate multiple people and their vital signs with high resolution and sensitivity. This time they validated their approach by measuring locations and vitals of up to five seated persons at a range of more than 1.5 m.

Some research stepped away from high-precision radars and rather focused on using cheaper omnipresent commodity devices, especially WiFi, in order to detect breathing. Wang et al. [42] proposed a system that employs channel state information (CSI) phase difference to estimate breathing rate using WiFi devices. They achieved low errors of under 2 breaths per minute. Abdelnasser et al. [43] developed a system using off-the-shelf WiFi devices, which is based on the received signal strength (RSS), achieving errors as low as 1 breath per minute and capable of apnea detection with high accuracy. Liu et al. [44] proposed another system that exploits fine-grained WiFi channel information to capture the minute movements caused by breathing and heart beats achieving low errors for both HR and RR.

With large recent successes of DL, some researchers focused on applying such methods to radar data. Bhattacharya et al. [45] proposed a DL approach for breathing and fall detection. A low frequency (sub 6 GHz) radar was used to localize a person and a small artificial neural network (ANN) model distinguished people from other moving objects via breathing. They managed to detect people with 95% accuracy and have shown a prototype fall detection system. Zhao et al. [46] attempted to recognize breathing disorders using a 2.4 GHz radar in a hospital setting. Temporal and frequency features were computed from the breathing waveform and several irregularities were classified between 67% and 95% accuracy using a support vector machine (SVM) classifier. Zhang [47] even proposed a heartbeat monitoring system, which relies on convolutional neural networks (CNNs) trained on features from electrocardiogram (ECG) and radar data. It was then evaluated only on the radar data, achieving over 90% accuracy, hinting at potential of transfer learning.

### 1.2. Critical Review and Context of Our Study

The area of contact-free sensing is broad, as seen from the related work, however, it is important to understand different sensor options as to be able to discern the advantages and disadvantages of each, and understand what are the reasons to use RF-based methods instead of optical alternatives. The pros and cons of each are summarized below.

#### 1.2.1. Optical Methods

- Advantages: These methods achieve state of the art results, with errors as low as 1 beat per minute (BPM) in HR estimation and under 1 breath per minute in breathing estimation. They allow for the measurement of skin perfusion (blood absorption), which in turn enables SpO2 (blood oxygen) measurement, which cannot be achieved with RF-based methods. Additionally, high resolution cameras offers rich context information about the user (activity, interactions, etc.) and allow for monitoring of several people at once using a single sensor, which is impossible with traditional contact sensors, as each person must be equipped their own device. Finally, optical sensors are readily available to almost everyone within smartphones, making them portable and available almost everywhere at any time.
- Disadvantages: Such methods require precise identification of ROI and relevant pixels for PPG reconstruction, which in turn allows for HR and RR estimation. Unwanted noise is introduced with facial hair, glasses, makeup, etc., which can make the process of skin segmentation more difficult. Furthermore, it is difficult to robustly detect skin with substantially different tone (melanin amount). This in turn requires specialized algorithms which bring additional computational load. A major challenge also lies in the common and quick changes in illumination and exposure. A sudden variation such as turning an additional light on or off can introduce a large variation in the reconstruction waveform, distorting it heavily. The motion-based methods alleviate some of these problems, but are in turn very sensitive to movement artefacts, since minute movements related to HR and RR must be identified. Finally, cameras can pose privacy concerns to many users, as it is common for people to be reluctant on being recorded continuously, in order to preserve privacy and anonymity as much as possible.

#### 1.2.2. RF-Based Methods

- Advantages: A major advantage of these methods is that they can be used to extract cardiorespiratory information even with almost total occlusion, for instance when a subject is heavily clothed and a facial ROI cannot be easily detected. The information can also be extracted at larger ranges of up to 30 m, given suitable radar with enough power and high frequency range. Of course privacy preservation is also a major advantage, as only the displacement of the chest or abdominal area is being captured rather than the frames containing facial images of a person.
- Disadvantages: The main limitation is the fact that only motion-related information can be captured, meaning no blood absorption information and no context. Additionally, similar to motion optical methods, these are prone to artefacts introduced by movement. Random body motion can be problematic [48] as well as potential self-motion of the radar itself [49]. Despite showing potential for long-distance monitoring, the waves also degrade due to so called free space loss, which causes the signal-to-noise ratio (SNR) to decrease with distance. Finally, specialized hardware is required, which is not as widespread as cameras, which are nowadays available in every mobile phone.

From the above we can conclude that RF-based monitoring comes with its set of challenges, but offers important advantages over optical methods (privacy preservation, robustness to occlusion, etc.), while also being less researched, especially in terms of detecting more complex states, not just respiration, so it is the focus of our study. In our study we did not estimate HR and RR directly, but rather focused on an end-to-end DL classification of more complex scenarios, where HR and RR are being purposefully influenced. We did this using traditional contact sensors (e.g., ECG) as well as a radar, which are available in a relatively new publicly available dataset. To the best of our knowledge, such an end-to-end classification without intermediate computation of HR or RR has not been done before, neither on this nor another similar dataset. It thus represents a baseline study for this dataset, as well as a performance comparison between traditional contact and privacy-preserving RF-based radar data input. Additionally, a multi-modal fusion (in terms of inputs) is investigated for potential improvements. Finally, we made our code open-source in order to offer the community a starting point for further work on these data. This is important in light of crisis of reproducibility in science [50], as well as from the view of available ANNs for use with radar data, as we did not find many publicly available ANNs for this domain.

## 2. Materials and Methods

Datasets for contact-free monitoring with labelled high-quality data have been scarce. Schellenberger et al. have addressed this gap by making publicly available a dataset of clinically recorded radar vital signs with synchronised reference sensor signals in 2020 [51]. We used this dataset as the basis of our study.

### 2.1. Dataset Description

The data were collected in a controlled setting by physicians at the Department of Palliative Medicine at the university hospital Erlangen. 30 healthy volunteers were strapped to a tilt table and participated in the following 5 scenarios, which always begun after an initial 10 min relaxation phase (lying down, calm breathing, minimal movement):

1. Resting: After the relaxation phase, the subject continued to lie relaxed with calm breathing, without any major movement.
2. Valsalva maneuver: In this scenario, the Valsalva maneuver is performed 3 times with pauses in between. The maneuver is defined as a forced expiratory effort against a closed airway [52], lasting for 20 s. After completion, the subject exhales and continues breathing calmly. There is a 5 min recovery period in between each of the three runs.
  This maneuver causes complex cardiovascular response, reflecting in the blood pressure (BP), HR and obviously RR, allowing for detection of abnormal responses in patients with different medical conditions [52].
3. Apnea: In this scenario the subjects held their breath twice for as long as possible. In the first simulation, the subject inhaled completely before holding the breath while in the second simulation the subject exhaled completely before holding breath.
4. Tilt up: Here the tilt table was raised from horizontal to vertical position, which leads to changes in BP and HR. The measurement starts in a horizontal position, then the table is slowly raised to 70° and the measurement is continued for 10 min.
  Full-body tilt at slow speed also elicits a cardiovascular response, however the severity of it depends on the subject.
5. Tilt down: This is the opposite of the previous scenario. The upright table is lowered down to the starting horizontal position and the measurement is again continued for 10 min.

Data was collected from 14 male and 16 female test subjects, with an average age of 30.7 ± 9.9 years and an average body mass index (BMI) of 23.2 ± 3.3 kg/m^2^. In total there were roughly 24 h worth of data, but not all scenarios were recorded for all people. Some summarization metadata is given in Table 1. It is important to note that this distribution is according to the original data where a whole scenario session was marked with the same label, even if only parts of it were the actual simulations. For instance, people only performed the Valsalva maneuver three times, each repetition lasting for 20 s, with 5 min recovery periods in between. However, the whole such recording session was marked as “Valsalva”, even though the recovery period is likely something between “Valsalva” and “Resting” in terms of physiological state. We addressed this problem and talk about it more in the following sections, also revisiting the distribution of data after this is taken into account.

A radar system based on Six-Port technology has been extended into a portable radar system and a bistatic antenna design was used to improve signal quality. The inclination angle of the antenna beams was ±10° for transmitting (Tx) and receiving (Rx) antenna, respectively, with a focal point at 40 cm. It was a 24 GHz continuous wave radar that was put in front of the subject chest. Technical implementation details of the recording setup are out of the scope of this paper, but we refer the reader to the original papers [51,53].

A movement in front of the antennas causes a measurable phase change Δϕ between Tx and Rx signal, which can be converted into a displacement change Δx with the known wavelength λ of the Tx signal using the following equation:

(1) $$\Delta x=\frac{\Delta \phi }{2\pi }\cdot \frac{\lambda }{2}$$

The radar does not capture the displacement changes (distance) directly, but rather two raw signal components I and Q are initially digitized simultaneously using a 24 bit analog-to-digital converter with a sampling frequency of 2000 Hz. The I and Q signal are used to calculate Δϕ by arctangent demodulation after an initial compensation for nonidealities, called ellipse reconstruction, is made [51,54].

In addition to the radar system, a reference system was also used to record precise contact signals with electrodes placed on the upper body of each subject and cuffs placed on upper hands and fingers. The Task Force Monitor (TFM) 3040i from CNSystems Medizintechnik GmbH was used to record the following signals [51]:

- Impedence (Z0): Electrical resistive impedance changes in relation to pulmonary air volume were used to measure respiration.
- Electrocardiogram (ECG): A three-channel ECG was used to record electrical activity of the heart during scenarios.
- Impedence cardiogram (ICG): ICG provides insight into the impedance change of the thorax by applying alternating small current between two electrodes on the body.
- Blood pressure (BP): The TFM enables continuous non-invasive BP measurement called Continuous Noninvasive Arterial Pressure (CNAP), which is measured by combining the measurement of an oscillometeric BP cuff and a cuff at the fingers measuring vascular unloading [55].

**Figure 3:**
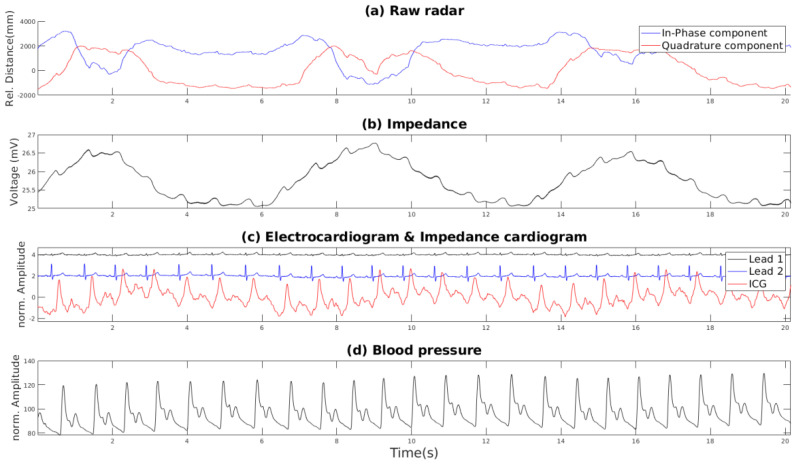
An example segment showing the initial raw signals in the dataset captured during a resting scenario. (**a**) shows the raw radar I and Q components, (**b**) shows the impedence, (**c**) shows the cardiograms, and (**d**) shows the blood pressure.

**Figure 4:**
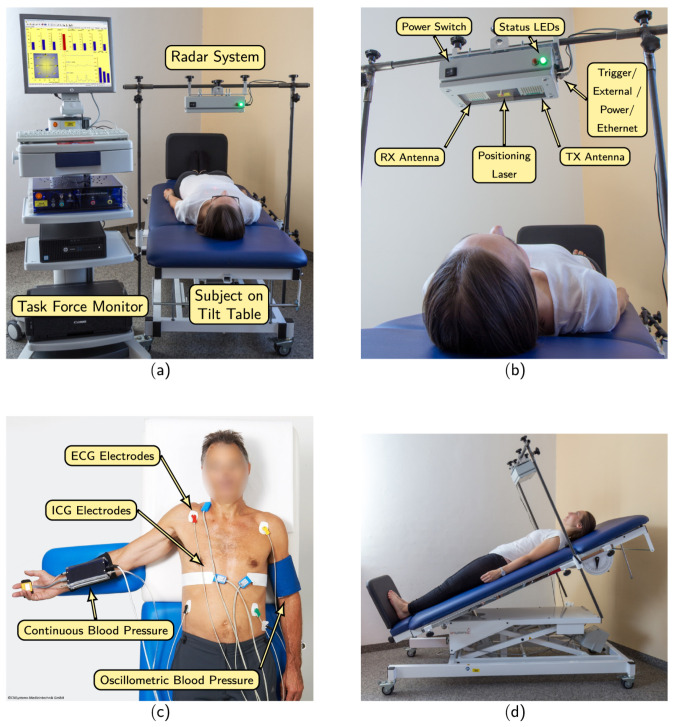
The recording setup which was used to collect the data. (**a**) Full system including reference TFM, radar setup and tilting table. (**b**) Details of the radar module. (**c**) Reference signals collected with contact sensors. (**d**) Subject in one of the tilting table scenarios. Credit to Schellenberger et al., used with their permission [51].

An example 20-s period of these raw signals is showin in Figure 3 and the recording setup is shown in Figure 4.

Even though only 5 signals were recorded in raw format, the authors of the dataset provide MATLAB scripts that allow for computation of several additional derived signals, for instance the distance signal is computed from the raw I and Q components of the radar. The distance can in turn be used to estimate cardiorespiratory waveforms. Similarly, the raw radar components can be used to compute an estimated signal of heart sound captured by the radar. This gives us potential additional data sources to be used in machine learning (ML).

### 2.2. Data Preparation and Preprocessing

Since the raw data are collected with high native sampling frequencies of up to 2000 Hz in continuous sessions, and since labels are provided for the whole session in the form of button presses (which signify important events in a scenario) we had to undertake a number of preprocessing steps to make it more suitable for ML. These include computation of derived signals using the scripts provided by the dataset authors, downsampling of all signals to a lower unified sampling frequency, band-pass filtering, segmentation into shorter overlapping windows, correct labelling based on the accompanying button-press signals and finally oversampling of minority class instances due to heavily imbalanced data.

#### 2.2.1. Computation of Derived Signals

As mentioned previously, one of the crucial starting steps was to obtain the distance measurement from the raw radar I and Q components to obtain cardiorespiratory information via methods provided by the original paper authors. Using the previously described raw signals, the following derived signals were computed with them:

- Distance: Distance computed from the raw I and Q radar components.
- Radar respiration: Respiration based on the distance computed previously.
- Heart sound: Heart sound based on the raw radar components and/or distance.
- Pulse: Heart pulse based on the heart sound computed previously.
- Contact respiration: Respiration based on the impedence recorded with the contact sensors.

**Figure 5:**
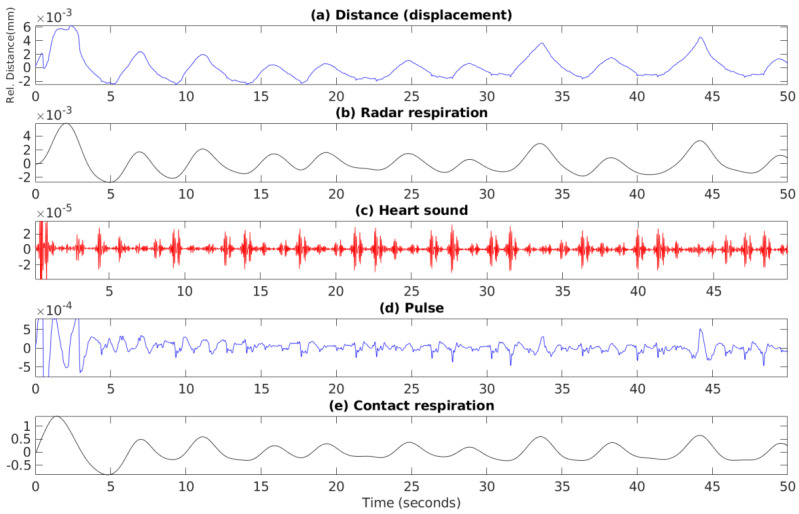
An example segment showing the derived signals in the dataset computed from the raw signals via scripts provided by the original paper authors. (**a**) shows the raw distance or displacement of chest as computed from the I and Q radar components, (**b**) is the smoothed version of (**a**), (**c**) shows the heart sound estimation from the radar data, (**d**) shows an approximation of heart pulse computed from (**c**), and (**e**) shows the contact reference respiration. Note that some transitions or computations of these derived signals are part of proprietary code and the source code is not disclosed by the dataset paper authors.

The distance computation details are described previously, but some details are not known on the computation of these derived signals due to the fact that the source code is not available, as it is proprietary [51]. However, based on the visual inspection of these signals shown in Figure 5, we can assume that the radar respiration and contact respiration are simply a filtered version of the distance and impedance signals.

#### 2.2.2. Downsampling and Filtering

The original data are sampled with relatively high and varied sampling frequencies from 100 Hz (impedance) to 2000 Hz (radar). As the data are plentiful and physiological information is not contained in high frequencies, we initially downsampled the signals to a unified lower sampling frequency of 100 Hz. We have chosen a rather safe value based on past experience, still allowing for some high-frequency components to be retained, which can be relevant for the ECG, but not really for other signals. We did this using the built-in MATLAB *resample()* function, which applies a final impulse response (FIR) Antialiasing Lowpass Filter to the input and compensates for the delay introduced by the filter [56].

Additionally, the data were filtered using a 4th order Butterworth band-pass filter with signal-specific cutoff frequencies. The lower cutoff was usually set at 0.1 Hz to remove the baseline drift while the upper cutoff ranged from 1 Hz for respiration-related signals to 20 Hz for the ECG, as the latter may contain information in the higher frequencies.

#### 2.2.3. Segmentation and Labelling

Recording session data are continuous and last from a few minutes to tens of minutes, depending on the session and scenario. We used a rather wide-spread and standard approach of first windowing the signal into shorter overlapping windows, which represent instances in our ML pipeline. The window duration and overlap amount are commonly discussed parameters and should be set correctly to capture the relevant information in each window. We opted for window length of 20 s in order to capture a few breathing cycles and the corresponding frequency information. Additionally we used a standard 50% overlap between windows, which increases the number of instances but still keeps them varied. Finally, we also normalized all the input data to the same [0, 1] range, since this is common practice that helps with the learning procedure, as the model weights must be updated on the same scale every time rather than being orders of magnitude apart.

**Figure 6:**
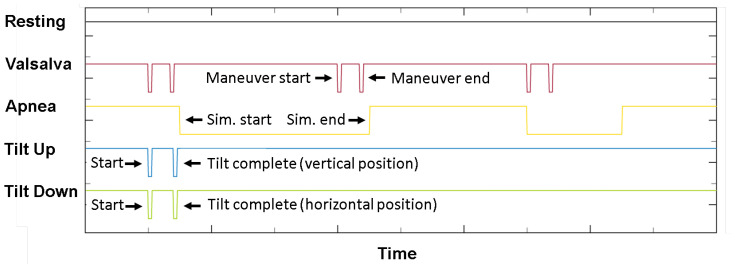
An example showing the button presses in the electrical signal and their meaning for each scenario (in majority of cases). Figure adapted from the original paper by Schellenberger et al. [51].

After the signal data are windowed, a corresponding class label must given to each instance. This is not completely trivial due to the fact that a whole recording session is marked with a single label (e.g., “Apnea” or “Valsalva”), but not all the signal data actually corresponds to this class. Instead, in some cases only a minority of the signal data corresponds to the actual marked scenario, like in the Valsalva scenario, where the maneuver is done on the scale of seconds or tens of seconds, while the recovery period is on the scale of minutes. A continuous electrical signal is provided in which button presses are identified by sharp drops in the signal amplitude. An additional concern is the fact that in a specific scenario the number of button presses in not always the same and subsequently the meaning of button presses changes as well—this is a consequence of the authors choice to change the number and meaning of buttong presses in later recording sessions compared to the early ones. A button was typically pressed at the start and end of the Valsalva maneuver and at the start and end of the tilting motion. For apnea however, the button was pressed and held throughout the simulation. Illustration of presses for each scenario is shown in Figure 6.

Using the button presses described previously, we assigned a class label to each window. The periods in which scenarios were actually performed were labelled as the actual scenarios while the periods in which the activity was ambiguous were initially labelled with as “Other”.

After all the preprocessing steps, segmentation and labelling were complete, the new distribution of instances in terms of class is more heavily skewed than initially, as shown in Table 2. Most importantly, the number of instances for “Valsalva” is actually much lower (closer to “Apnea”) due to the long recovery periods being put into the “Other” class.

#### 2.2.4. Oversampling

The heavy imbalance in the data is troublesome since ML algorithms have difficulties learning from just so few instances of some compared to other classes. Furthermore it is difficult to evaluate robustly when some classes are so heavily underrepresented. Naturally we wanted to keep as much data as possible, especially since we opted to focus on DL approaches where more data are especially valuable, so we did not wish to undersample the data to fix the imbalance. Instead we decided to oversample the minority classes using the Synthetic Minority Oversampling TEchnique (SMOTE) [57]. The method synthesizes new unique instances of the undersampled class by selecting a random instance and then finding *k* of its nearest neighbours. A randomly selected neighbour is then chosen and a synthetic instance is created at a randomly selected point between the two examples in the feature space [57]. This creates plausible new instances which differ from the original ones, especially if *k* is sufficiently large.

We used this method to equalize the distribution of all classes, but most importantly to oversample the “Valsalva” and “Apnea” class, so that we had enough instances in the training data to allow the model to learn some characteristics of these classes and not just ignore them due to their low representation and influence on the accuracy.

### 2.3. Experimental Setup

As we discussed previously, the input instances *X* to our ML pipeline were the 20-s windows of the signals, some originating from the radar sensor (denoted as “radar” from here on) and others originating from the contact TFM device (denoted as “contact” from here on). We thus had two modalities, radar and contact, the former consisting of six total signals (I, Q, distance, respiration, heart sound and pulse) and the latter also consisting of six total signals (blood pressure, two ECG leads, ICG, impedance, respiration). These initial instances are temporal, however, we also wanted to capture some frequency information. Thus, we also computed the squared absolute value of the FFT of each instance, which gave us the information about the frequencies present in each instance. The frequency spectrum representation does not include the phase information, which is still present in the temporal part. This multiplies the number of inputs by two, having a temporal and frequency representation of each instance for each signal, but we can only take half of the frequency representation, as the FFT is mirrored.

For our target variable *Y*, we initially had six classes as defined in the preprocessing part, but we decided to remove the “Other” class for evaluation, since it is semantically not clear what happens in those periods, as influences of several other classes can be present. Thus, we conducted our experiments with five classes defined in the original dataset, which were one-hot encoded.

In order to evaluate the classification performance of our models, a robust evaluation experiment for DL was designed. First, a classification metric and loss function were chosen, which in our case were the categorical accuracy and categorical cross-entropy. This pair is commonly used in cases where one-hot encoding is used for labels, which is common practice when multi-class classification is being done. Then the data of all subjects was stacked, ensuring the temporal order was kept and data of each subject remained together. This is important for the subsequent splitting, as it may happen that in case of shuffling the internal results are overly optimistic, as overfitting happens due to similar instances (of a single subject) being present in both training and validation data. Then a stratified 5-fold cross validation (CV) experiment was done, in which 80% of the data are always taken for training and 20% is withheld for testing, ensuring balanced class distribution in both training and testing data. This is repeated five times, ensuring that each part of the data is independently tested on once, and the results are then averaged across all folds. As we focused on DL approaches, we additionally split the training data into internal training and validation, again in 80–20% ratio. The models were trained on the internal training data, and the purpose of validation data was to guide the model convergence and control overfitting to the training data, with the aim of achieving generalization capability of the model, which was then evaluated on the left out testing data.

This sort of k-fold CV is relatively robust with the exception of the border instances where the split is made. The split makes it so that some neighbouring instances of a single subject are put into train and test set, probably causing an overoptimistic performance for those instances. In 5-fold CV this happens five times. In the first and the last fold, data of a single subject is split, while in the intermediate folds data of two subjects are split. However, each pair of subsequent intermediate folds shares one such subject. In total this brings the number of subjects whose data gets split to 1–2 on average in each evaluation fold. However, all 30 subjects are eventually evaluated on and in aggregate only distinct 4 subjects data gets split. Furthermore, as data gets internally split again into training and validation, this further dilutes the number of problematic instances in the actual model training. When several tens of subjects and several tens of thousands of instances are being considered, this influence is minimal and does not notably effect the results. A way to circumvent this is to instead use the leave-one-subject-out (LOSO) evaluation setup, where all but one patients are used for training and the left out patient is used for testing, without any neighbouring instances appearing in both sets. The downside of the LOSO setup is that a model has to be trained and evaluated many more times compared to a k-fold CV in cases where number of subjects is notably greater than k. This is especially impactful in DL, where the amount of data is typically large and the model training can take quite some time and require a lot of computational power.

### 2.4. Deep Learning Architectures

Due to the plentiful data and recent successes of DL, we decided to investigate ANNs to build a classification model. The main advantage of such an approach compared to traditional ML is that with an end-to-end design the signals can be input directly into the model, without the need for explicit feature engineering and extraction, as this is done implicitly by the network itself.

We decided to use an independent-branch approach, where a branch of an ANN is built for each of the input signals, the branches are then concatenated, and additional learning layers are put on top of it. Fully-connected networks were designed in three major variants based on the input modality:

1. Contact network: A variant where a branch is built for each of the contact sensors
2. Contact-free network: A variant where a branch is built for each of the radar sensors
3. Fusion network: A variant where branches are built for all of the available input sensors, regardless of sensor modality

We focused on the fully-connected architectures for several reasons. First and foremost, they performed better compared to CNNs and long-short-term-memory networks (LSTMs) in preliminary experiments—the latter two had problems learning at all since the loss and accuracy did not converge, potentially due to too many parameters and the vanishing gradient problem. Additionally, CNNs and LSTMs have additional hyperparameters to investigate, further increasing the search space and computational cost of the experiments.

**Figure 7:**
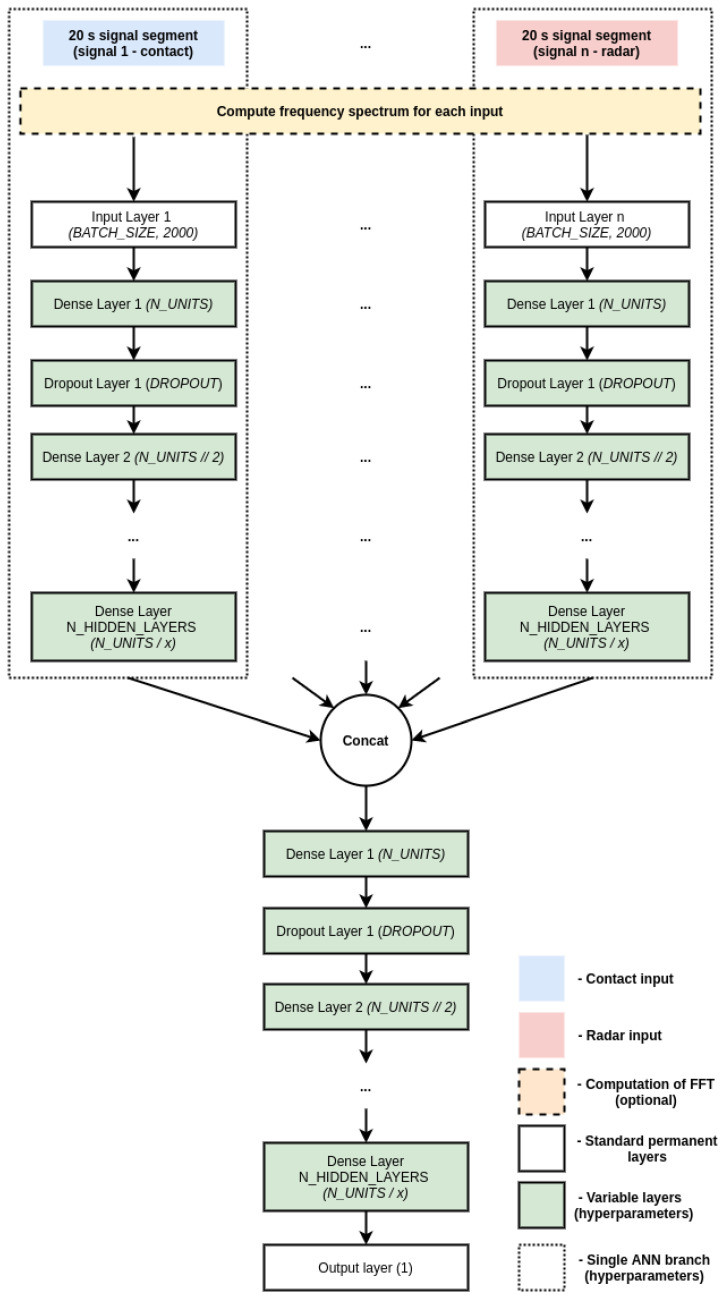
Conceptual schematic of a the branched ANN architectural paradigm investigated in our work. There are different possible input modalities (contact, radar or fusion) and data types (temporal, frequency/FFT or fusion) and different possible hyperparameters.

As mentioned previously, the inputs into the ANN can also be in either temporal (actual segments of the signals) or frequency domain (squared absolute value of the FFT of a given segment), or a combination of both. This means notable variation in the possible inputs and variations of the network architectures. A general schematic of the investigated branched architecture alongside some relevant hyperparameters is shown in Figure 7. All investigated models follow this paradigm, but differ slightly in the architecture, hyperparameters and inputs, so we show a single generalized scheme for brevity.

Since we have many branches, the number of parameters of the architectures is inherently large, however, this can limit the generalization capability and increases the computational cost of training. We thus decided to limit the depth of the network and use suitable dropout mechanisms, dropping some random connections.

This brings us to the investigation of the hyperparameters of the ANN models. It is known that the hyperparameter space of neural networks is large and it often comes down to researcher’s experience and intuition to narrow down the space and find a good set. We employed a systematic approach—a list of hyperparameters and the values to investigate were initially defined as given in Table 3 and then a random search, which included running the 5-fold CV experiment on a randomly selected set and repeating this *n* times, was conducted. The search was done separately for contact, contact-free and fusion networks, for temporal and frequency inputs. Naturally, the computational cost of such an experiment is quite large, so we limited the initial options and then further narrowed it down based on performance. The search and training were conducted on a workstation with 32 GB of RAM and nVidia Quadro P6000 GPU with 24 GB of GDDR5 vRAM.

## 3. Results

We compared different input modalities and ANN architectures in terms of their average categorical accuracy in the 5-fold CV experiment described earlier. The initial baseline of the majority vote classifier is at 20% accuracy, due to balanced data and having 5 classes. Additionally, we considered classification performance on per-class basis via inspection of confusion matrices in the same experiments. Numerical results are reported in Table 4.

**Figure 8:**
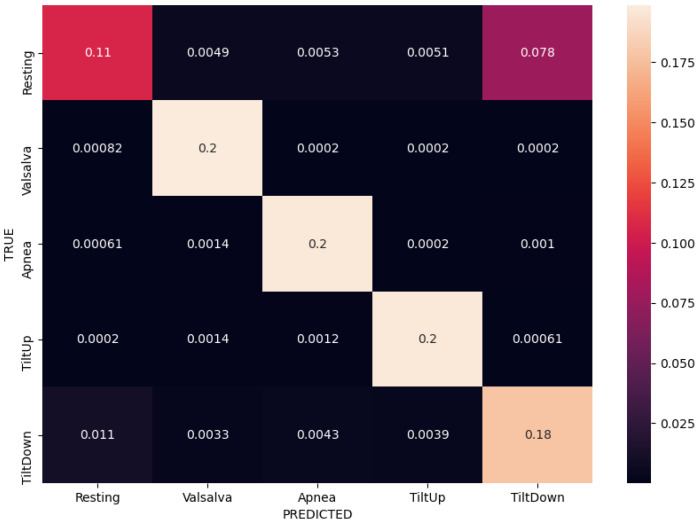
Normalized confusion matrix for contact modality using FFT inputs in a fully-connected ANN, achieving 88% accuracy.

**Figure 9:**
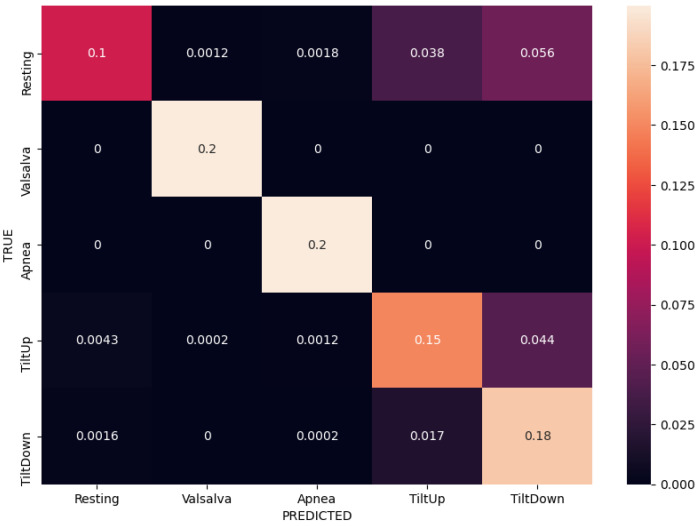
Normalized confusion matrix for radar modality using FFT inputs in a fully-connected ANN, achieving 83% accuracy.

**Figure 10:**
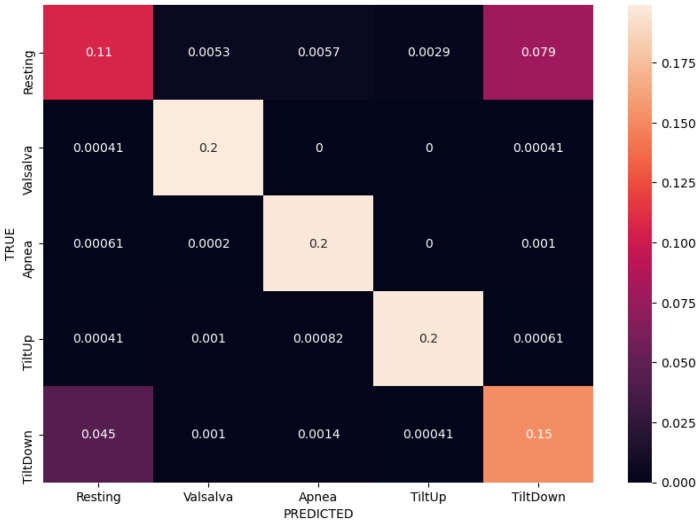
Normalized confusion matrix for sensor fusion using both temporal and FFT inputs in a fully-connected ANN, achieving 88% accuracy.

Normalized confusion matrices corresponding to the best results (bolded) for each of the three input modalities are shown in Figure 8, Figure 9 and Figure 10. Matrices are created in each fold separately during the experiments, but these are the average of all the per-fold matrices in a given experiment. We can see that similar results can be achieved using either temporal or frequency or combination of inputs, as the network is apparently able to model the relationship between either the actual waveform and class, or the frequencies in the waveform and class and relevant information seems to be present in both. The results are quite similar, so the confusion matrices are shown with up to 4 decimal places.

These results were obtained using fully-connected ANNs with corresponding optimized hyperparameter sets given in Table 5.

**Figure 11:**
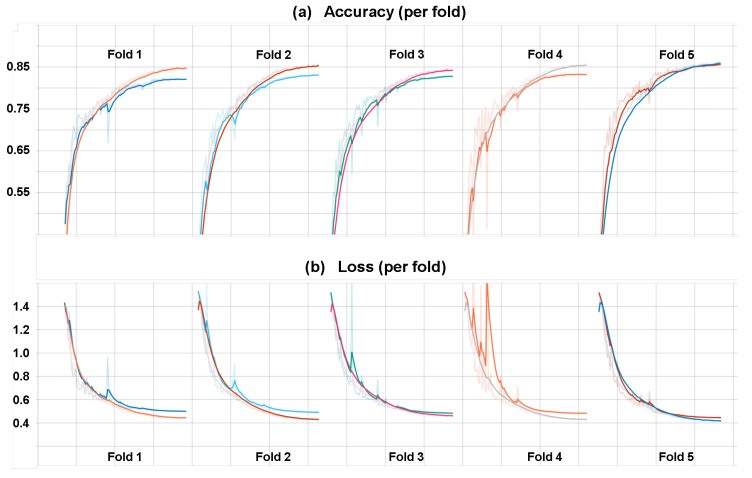
(**a**) shows pairs of train and validation accuracies for each fold in one of the best-performing 5-fold CV experiments over 100 epochs. (**b**) shows pairs of train and validation losses for each fold in one of the best-performing 5-fold CV experiments over 100 epochs. Darker lines are slightly smoothed versions of the originals.

One of the important things to consider during training is the increase and convergence of the categorical accuracy and corresponding decrease and convergence of the categorical cross-entropy loss. This shows the capability of the model to learn, however, this learning is always done on the training data, which is fed to the network many times over many epochs in order to adjust the weights accordingly via back-propagation. This is where the validation data comes into play, as the model can in theory (given enough expressive power via the number of weights) overfit completely to the training data, but as a consequence performs very poorly on previously unseen data. A separate validation set controls the performance of the model on unseen data, ensuring that it is generalizing appropriately, which is reflected in the same behaviour of accuracy and loss on both the training and validation data. Example of this behaviour in one of our 5-fold CV experiments is shown in Figure 11, where we can see the increase of accuracy for both training and validation up to 0.85 over 100 epochs, converging at roughly 70 epochs. This behaviour is similar across most best-performing experiments so we just show a single example.

## 4. Discussion

A very important aspect of DL where the networks generally have many parameters and large expressive power, is the generalization capability. We ensured strict separation of training and testing instances in our experimental design and noticed from the results that the performance was relatively consistent across all folds. We did not notice cases where a large variation in the accuracy happened between folds (e.g., one fold would have much lower accuracy compared to others, but the average would somewhat mask this), but rather a degree of stability was observed. This can also be seen in Figure 11, where we notice quite consistent accuraccy and loss curves across all folds.

Another concern with hyperparameter optimization is the possibility of model selection overfiting on a single set—meaning there would exist just a single set of hyperparameters that happens to work well on this specific case (data). This was again observed not to be the case, since top three best-performing hyperparameter sets for each experimental case achieved comparable accuracies within a few percentage points, indicating that several sets of hyperparameters exist that work well.

Taking a closer look at the confusion matrices we can consistently observe very good performance when classifying “Valsalva” and “Apnea” classes, while there are more missclassifications of “Resting”, “Tilt Up” and “Tilt Down” classes. This is somewhat expected as the former two classes are distinctly different in terms of breathing, while the latter three are more similar in terms of respiratory movement and thus more difficult to separate. We further notice that the model trained on the radar modality confuses the tilt up and down movements more, and these are also confused with resting sometimes. On the other hand the model trained on the contact modality only consuses one of the tilts with resting. The radar modality is intuitively less rich (as it contains only the movement information) so that model is likely to perform worse.

In terms of input modalities, we can conclude that the radar modality achieves somewhat worse results compared to contact sensors, but not by much. The superiority of contact sensors is expected, since they are less sensitive to movement noise and use different measuring principles (radar relies on movement only, while contact sensors can capture electrical signals, vascular response via cuffs, etc.), but the performance of the radar modality is still promising. The fusion of both modalities does not seem to bring much improvement based on our experiments, but does bring additional space and time requirements, as the number of branches doubles. Similar results are also observed in terms of different data types (temporal or frequency domain), where we did not observe superiority of one or another, which seems to indicate that relevant information is contained in both the temporal (waveform itself) and frequency (frequency spectrum) domain. Again, fusion of both data types did generally not bring notable improvements, but it once again doubles the number of ANN branches, increasing the size of the model further.

The hyperparameters of the ANNs majorly influenced the results of our experiments, as the worst sets performed as badly as the majority vote classifier and did not converge at all, while the best sets achieved quite decent results. This indicates that the architecture selection and hyperparameter tuning are vital when evaluating ANNs on this dataset.

### Limitations

An obvious limitation of our experiments is the fact that only fully connected ANNs were explored in detail, while some work reports success when 1D CNNs are used alongside long-short-term memory networks (LSTMs) [58]. Although we did attempt some initial training with such architectures, we did not include them in the final experiments due to initial poor results and very high time requirements.

Additionally, we did rely on some derived signals for our inputs, which require additional methods to obtain and present more computational cost. Further generalization would make sense in terms of using only the raw signal inputs, thus omitting the need for methods to compute derived signals and reducing the overall time complexity of the whole pipeline.

Finally we relied heavily on the assumption that ANNs are capable of internally computing good features and did not investigate traditional ML approaches, where features are manually crafted and then explicitly fed into an ML model.

## 5. Conclusions

To summarize, we investigated classification of complex hemodynamics scenarios from a publicly available dataset containing both contact sensors and radar recordings. We proposed a branched DL approach with a robust preprocessing and evaluation setup, and investigated the performance in terms of accuracy. We additionally conducted hyperparameter optimization and limited architecture search to improve the results. Combinations of three input modalities (contact, radar and fusion) and two data types (time and frequency domain) were investigated.

We showed that traditional contact sensors still achieve the best results, but accuracy when only using the radar modality is not far behind, indicating that contact-free sensing can achieve reasonably good results in healthcare monitoring even for complex scenarios, not just physiological parameter estimation. Additionally, an end-to-end approach was shown to be feasible, without explicitly computing HR or RR, as these can be modelled internally by ANNs. Fusing several modalities did not show notable improvements in our experiments, neither in terms of input modalities nor data types. Finally we showed that relevant information is contained both in the time and frequency domain, as ANNs can achieve good accuracies with either data type inputs.

In accordance with limitations of our study, future work includes investigation of additional DL architectures, especially CNNs and LSTMs, and investigation of using only raw signals without any additional derived signal computation. Recent trends in DL also suggest the usage of attention mechanisms, which can give performance improvements but also help with the explainability of the models, which is traditionally a challenge in DL. Additionally, the fusion of input sensor modalities is one way of exploration, as investigated in this paper, but another way is the potential fusion of complementary sensors in terms of their contributions—for instance ECG could be combined with camera or radar (if HR waveform is estimated) to measure pulse transit time and estimate BP in the future.

The main contribution of this research is the performance comparison of contact and contact-free data modalities and potential advantages (or lack thereof) of data fusion. Additionally it showed that reasonably high accuracy can be achieved for classification of more complex states (compared to just HR or RR) related to physiological changes when only radar sensor is used. This can be useful both in a home environment and a hospital setting, especially when privacy must be preserved or important time can be saved by not equipping each patient with a set of wearable sensors. The former is increasingly important, as seen by regulations such as General Directive of Privacy Regulation (GDPR) while the latter must also not be underestimated—ongoing pandemic of COVID-19 has shown that in case of hospital overloads, time saved on each patient is vital. Additionally, contact-free sensing eliminates the risk of contamination that exists if the same contact sensors (albeit sanitized) are re-used across patients. Furthermore, the comfort of contact-free monitoring is also very important, especially in patients who cannot equip wearables, for example patients with major skin burns or other skin conditions. Finally, this research represents a baseline classification study on a recently published publicly available dataset and since the code is made available at https://github.com/gslapnicar/hemodynamics-class-radar (accessed on 4 March 2021), it serves as a starting point for further research on this valuable dataset.

## Figures and Tables

**Table 1:** Some metadata describing the quantity and distribution of data per-scenario.

Scenario	Subject Count	Duration	Fraction of All Data
Resting	30	5.3 h	22%
Valsalva	27	7.8 h	33%
Apnea	24	1.3 h	5%
TiltUp	27	4.8 h	20%
TiltDown	27	4.8 h	20%

**Table 2:** Some metadata describing the quantity and distribution of windowed instances per-scenario. It is quite different both in duration and distribution from the original labels due to introduction of the label “Other” and overlapping windows.

Class (Integer Label)	Instance Count	Duration	Fraction of All Data
Other (0)	3021	16.8 h	36%
Resting (1)	1860	10.3 h	22%
Valsalva (2)	173	1 h	2%
Apnea (3)	184	1 h	2%
TiltUp (4)	1575	8.7 h	19%
TiltDown (5)	1630	9.0 h	19%

**Table 3:** The set of hyperparameters we investigated for our ANN models.

Hyperparameter	Investigated Values
N_HIDDEN_LAYERS	[1, 2, 3]
N_UNITS	[32, 64, 128]
ACTIVATION	[*relu*, *tanh*]
DROPOUT	[0.2, 0.3, 0.4]
LEARNING_RATE	[0.005, 0.01, 0.05]
OPTIMIZER	[*Adam*, *SGD*, *AdaDelta*, *RMSprop*]
REGULARIZER	[*l1*, *l2*]
REGULARIZATION	[0.001, 0.005, 0.01]
BATCH_SIZE	[64, 128]
N_EPOCHS	100
LOSS	*categorical_crossentropy*
LAST_ACTIVATION	*softmax*
INITIALIZER	*GlorotNormal*

**Table 4:** The best categorical accuracies achieved in the 5-fold CV experiment for different modalities and input types using an optimized (best-performing) fully connected ANN model.

	Temporal Data	Frequency Data (FFT)	Temp. + Freq. (FFT)	Mean
Contact	0.87	0.88	0.88	0.88
Radar	0.83	0.83	0.82	0.83
Fusion	0.87	0.87	0.88	0.87
Mean	0.86	0.86	0.86	

**Table 5:** The best best performing fully-connected ANNs with corresponding optimized hyperparameters.

	Best-Performing ANN [7]	Optimized Hyperparameters
Contact		N_HIDDEN_LAYERS: 1
		N_UNITS: 32
	Signal inputs: 6	ACTIVATION: *tanh*
	Temporal branches: 0	DROPOUT: 0.3
	FFT branches: 6	LEARNING_RATE: 0.005
	Concatination: 1	OPTIMIZER: *Adam*
	Output branches: 1	REGULARIZER: *l2*
		REGULARIZATION: 0.001
		BATCH_SIZE: 64
Radar		N_HIDDEN_LAYERS: 1
		N_UNITS: 64
	Signal inputs: 6	ACTIVATION: *tanh*
	Temporal branches: 0	DROPOUT: 0.3
	FFT branches: 6	LEARNING_RATE: 0.05
	Concatination: 1	OPTIMIZER: *Adam*
	Output branches: 1	REGULARIZER: *l2*
		REGULARIZATION: 0.001
		BATCH_SIZE: 128
Fusion		N_HIDDEN_LAYERS: 1
		N_UNITS: 32
	Signal inputs: 12	ACTIVATION: *tanh*
	Temporal branches: 12	DROPOUT: 0.4
	FFT branches: 12	LEARNING_RATE: 0.001
	Concatination: 1	OPTIMIZER: *Adam*
	Output branches: 1	REGULARIZER: *l2*
		REGULARIZATION: 0.001
		BATCH_SIZE: 32

## Data Availability

The data used in this research is publicly available at https://figshare.com/articles/dataset/A_dataset_of_clinically_recorded_radar_vital_signs_with_synchronised_reference_sensor_signals/12186516/2 (accessed on 4 March 2021).

## Abbreviations

The following abbreviations are used in this manuscript:

COVID-19	Coronavirus disease 2019
IR	Infrared
FM	Frequency modulation
UWB	Ultra wide band
CSI	Channel state information
RSS	Received signal strength
ANN	Artificial neural network
LSTM	Long-short-term-memory
EER	Equal error rates
IBI	Inter-beat interval
